# The Use of “Cancer Ratio” in Differentiating Malignant and Tuberculous Pleural Effusions: Protocol for a Prospective Observational Study

**DOI:** 10.2196/56592

**Published:** 2024-12-23

**Authors:** Sai Pooja Chalamalasetty, Preetam Acharya, Thomas Antony, Anand Ramakrishna, Himani Kotian

**Affiliations:** 1 Department of Respiratory Medicine Kasturba Medical College Mangalore Manipal Academy of Higher Education, Karnataka Manipal, 576 104 India; 2 Department of Community Medicine Kasturba Medical College Mangalore Manipal Academy of Higher Education, Karnataka Manipal, 576 104 India

**Keywords:** tuberculosis, pleural, pleural effusion, malignant, biomarkers, tumor, cancer ratio, differential diagnosis, respiratory disease

## Abstract

**Background:**

Differentiating between tuberculosis and malignancy as the cause of an exudative lymphocyte predominant pleural effusion is difficult due to similarities in the cellular and biochemical characteristics of the pleural fluid in both conditions. Microbiological tests in tubercular pleural effusions have a poor diagnostic yield, and the long turnaround time for results prevents an early diagnosis. The diagnosis of malignant pleural effusion (MPE) is hampered by a variable yield of pleural fluid cytology and closed pleural biopsy and the fact that thoracoscopy may not be readily available or feasible in each patient. A key gap in the existing knowledge is the performance of the serum lactate dehydrogenase to pleural adenosine deaminase ratio (ie, “cancer ratio”; CR) in differentiating between tuberculous and MPE in a high tuberculosis prevalence country like India, although its use has been well established in Western literature. The CR may find a practical application in the community health care settings in low-income countries without ready access to biopsy.

**Objective:**

This study aimed to evaluate the CR as a test to differentiate tubercular and malignant etiology in patients with an exudative lymphocyte predominant pleural effusion. Secondary objectives to be assessed include a comparison of CR to pleural fluid carcinoembryonic antigen in MPE and the association of histologic type of lung carcinoma to the CR positivity.

**Methods:**

This hospital-based, prospective, observational study will include patients admitted with pleural effusion whose pleural fluid reports indicate a lymphocyte-predominant exudate. The ability of the CR to discriminate between tuberculous and MPE will be evaluated as a primary objective of this study. The performance of CR and pleural fluid carcinoembryonic antigen in the diagnosis of MPE will be compared using the receiver operating characteristics and area under the curve for both tests as a secondary objective. The association between a positive CR and histologic type of lung cancer will be analyzed as well.

**Results:**

Data collection began in June 2022. As of March 24, 2024, we have recruited 22 patients. Outcomes of the study are expected at the end of 2024.

**Conclusions:**

The results of this study will provide an objective basis for the use of CR in differentiating between tuberculosis and malignancy as the cause of an exudative lymphocyte predominant pleural effusion.

**International Registered Report Identifier (IRRID):**

DERR1-10.2196/56592

## Introduction

Pleural effusion is a common mode of presentation in patients with both respiratory and systemic diseases. The global incidence of pleural effusion is estimated to be 5/1000 person-years, with 1.5 million new cases diagnosed each year in the United States alone [1]. Previously published studies in India have revealed tuberculosis and malignancy to be common causes of exudative pleural effusion in India [2-5]. The implications of tuberculosis and malignancy being the cause of pleural effusion are strikingly different. The diagnosis of tubercular pleural effusion (TPE) indicates a potentially curable disease. In contrast, a malignant pleural effusion (MPE) is evidence of an advanced stage of malignancy with incurability and a poor prognosis [6].

TPE occurs in approximately 5% of patients with tuberculosis infection. It results from a delayed hypersensitivity reaction to tuberculoproteins present in the pleural space, and hence, microbiological tests on the pleural fluid are generally negative. Acid-fast bacillus stain on the pleural fluid is positive in less than 10% of the samples, whereas pleural fluid culture for mycobacteria is positive in approximately 30%. Liquid culture media, such as the BACTEC-Mycobacteria Growth Indicator Tube–automated mycobacterial detection system, provide higher yields and faster results than conventional solid culture methods [7]. However, these microbiological tests for confirmation of TPE can be time-consuming, and the decision to initiate treatment would need to be taken much before this. Due to the shortcomings associated with the microbiological tests, pleural fluid adenosine deaminase (ADA) has been commonly used as a surrogate biomarker to establish the diagnosis of pleural tuberculosis. ADA is a T lymphocyte enzyme elevated in diseases like tuberculosis, where cellular immunity is stimulated [8]. Almost all patients with TPE have a pleural fluid ADA level above 40 U/L—the most widely accepted cutoff value for the diagnosis of TPE, at which it has a sensitivity and specificity of 92% and 90%, respectively [9]. Using the GeneXpert Mycobacterium tuberculosis/Rifampicin Ultra assay on the pleural fluid sample is not recommended for the diagnosis of TPE, as the sensitivity of this test on the pleural fluid is very low, leading to either a missed or delayed diagnosis [10].

Approximately 15% of all patients with cancer develop MPE [11]. The diagnosis of MPE is made by demonstrating exfoliated malignant cells in the pleural fluid or histopathological evidence of malignancy in the pleural tissue obtained by either a thoracoscopic or a closed pleural biopsy. Pleural fluid cytology has an overall sensitivity of 58.2% (range 20.5%-86%), with the yield depending on factors like the mechanism of effusion, type of primary tumor, number of specimens, and skill of the cytopathologist [12,13]. The diagnostic yield of closed pleural biopsy in MPE ranges from 39% to 75%, which is inferior to cytology since the blind procedure may sometimes yield insufficient or a nonrepresentative sample [8]. Thoracoscopic evaluation and biopsy is the investigation of choice where the initial pleural fluid reports are inconclusive for MPE. However, thoracoscopy is an expensive procedure needing expertise and a thoracic surgery backup, and its availability remains limited in low-income countries. In this context, tumor markers in the pleural fluid have been evaluated as a potential tool for diagnosing MPE. A study evaluating the diagnostic value of tumor markers reported pleural fluid carcinoembryonic antigen (CEA) as having the highest diagnostic accuracy with a sensitivity of 72.2%, a specificity of 92.2%, a positive predictive value of 89.7%, and a negative predictive value of 77.8% [14]. A study involving 1230 patients also revealed pleural fluid CEA (using a cutoff value of 3.7 ng/mL) to be the best diagnostic marker in separating pleural effusions of malignant from benign etiology [15]. Krishnan et al [16], in their study, showed the sensitivity and specificity of pleural fluid CEA in diagnosing MPE to be 93.5% and 73% while using a cutoff value of 2.15 ng/mL. Other studies have shown varying sensitivity and specificity of pleural fluid CEA in MPE diagnosis, which could be attributed to the varying CEA cutoff values in these studies, which ranged from 3 to 50 ng/mL [16-20]. The wide range in the cutoff values and the observation that false positive results for pleural fluid CEA are also seen in parapneumonic effusion, empyema, and TPE have limited the clinical use of this test [21].

Verma et al [22] first described the use of the “cancer ratio” (CR), that is, the serum lactate dehydrogenase (LDH) to pleural adenosine deaminase ratio, to identify MPE and reported a high specificity and sensitivity using a cutoff level >20. LDH is a cytoplasmic enzyme present in tissues involved in anaerobic glycolysis and gluconeogenesis. High LDH levels are seen in myocardial infarction, acute liver failure, hemolytic anemias, myopathies, sepsis, and cancers. The increase in serum LDH in cancers is due to the preferential use of glycolysis for energy in the tumor cells, which is mediated by LDH [23]. A high ADA level in pleural fluid suggests TPE, whereas a low ADA level indicates a high probability of an MPE. It is postulated that combining both markers as a ratio has the potential for use in diagnosing MPE. Both serum LDH and pleural fluid ADA are included in the routine laboratory investigations in patients with pleural effusion, making the CR a simple, point-of-care test without any additional cost to the patient.

The use of CR in the diagnosis of both neutrophil- and lymphocyte-predominant exudative MPE has been established in a previous study [22]. Pooled data from a meta-analysis of 7 studies showed an overall sensitivity of 0.96, a specificity of 0.88, a positive likelihood ratio of 7.70, and a negative likelihood ratio of 0.05. The area under the curve was 0.98, indicating a high overall diagnostic accuracy for CR in the prediction of MPE [24]. Other studies, while validating the use of CR in diagnosing MPE, have suggested measuring “CR plus” (ie, the ratio of CR to the percentage of differential pleural fluid lymphocyte count) to improve the specificity of the CR in differentiating MPE from TPE [25,26].

The etiology of pleural effusion remains unknown in up to 5% to 25% of patients after the initial diagnostic workup. The important conditions to consider in patients with recurrent undiagnosed pleural exudates are malignancy and tuberculosis [27]. CR has been reported in the Western literature as a useful test on the pleural fluid sample that could quickly and reliably separate MPE from TPE. This study aims to explore its use in the Indian population with a high prevalence of tuberculosis. Also, there is no literature studying the association of a positive CR with the histologic type of cancer and limited studies comparing the performance of pleural fluid CEA and CR estimations in diagnosing MPE, 2 questions the authors seek to answer through this study.

## Methods

The key aspects of the study protocol are summarized below.

### Study Design and Setting

This study is designed as a hospital-based, prospective, observational study. This study will be conducted in the teaching hospitals affiliated with Kasturba Medical College, which includes Kasturba Medical College Hospital, Attavar, Mangalore, and Government Wenlock District Hospital, Mangalore, located in the southern state of Karnataka in India.

### Study Objectives

#### Primary Objective

The primary objective was to evaluate the CR as a test to differentiate tubercular and malignant etiology in patients with an exudative lymphocytic pleural effusion.

#### Secondary Objectives

Secondary objectives were (1) to compare the performance of CR and pleural fluid CEA estimations in diagnosing MPE and (2) to determine the association of histologic types of lung cancers, that is, squamous, small-cell, adenocarcinoma, and large-cell carcinoma, to the CR positivity in MPEs.

#### Eligibility Criteria

This study will be conducted among patients of both genders, aged 18 years and older, admitted into the teaching hospitals affiliated with Kasturba Medical College over 2 years from 2022 to 2024 for the management of pleural effusion and are planned for a pleural fluid aspiration and analysis. The study inclusion and exclusion criteria are specified in Textbox 1. The variables and the operational definitions used in the study are specified in Textbox 2.

**Inclusion criteria and exclusion criteria.**

**Inclusion criteria**

- Patients admitted with pleural effusion whose pleural fluid reports are suggestive of:
  An exudative pleural effusion as per the Light [8] criteria, that is, meeting 1 or more of the following criteria:
    Pleural fluid protein: serum protein >.5
    Pleural fluid lactate dehydrogenase: serum lactate dehydrogenase >.6
    Pleural fluid lactate dehydrogenase is more than two-thirds of the normal upper limit for serum
  Pleural fluid is lymphocyte predominant (>50% lymphocytes in pleural fluid differential cell count)

**Exclusion criteria**

- Aspirated pleural fluid is a transudate as per the Light [8] criteria
- Aspirated pleural fluid is an exudate having neutrophil predominance (>50% neutrophils in pleural fluid differential cell count)
- Lymphocyte predominant (>50% lymphocytes in pleural fluid differential cell count) exudative pleural effusion of etiologies other than tuberculosis or malignancy

**Variables and operational definitions.**

- A diagnosis of malignant pleural effusion will be considered based on criteria that consist of:
  Pleural fluid cytology positive for malignant cells, or
  Pleural biopsy histopathology is suggestive of malignancy
- A diagnosis of tuberculous pleural effusion will be considered based on the following:
  Pleural fluid acid-fast bacilli positive on Ziehl-Nelsen stain, or
  Mycobacterial growth on pleural fluid culture, or
  Pleural fluid GeneXpert Ultra: Mycobacterium tuberculosis and rifampicin resistance detection, (Cepheid) positive for M tuberculosis, or
  Pleural biopsy specimen reported as tuberculous etiology, or
  Pleural fluid adenosine deaminase value of >40 U/L with negative malignant cytology in an exudative lymphocyte predominant effusion followed by a decision of the clinician to treat with anti-tuberculous drugs [8]
- A cancer ratio (serum lactate dehydrogenase to pleural adenosine deaminase ratio) at a cutoff level of > 20 will be used for identifying malignant pleural effusion
  The prespecified threshold of 20 was based on a previous study, which showed a sensitivity of 0.84 and a specificity of 0.92 when using this cutoff [15].
- A pleural fluid carcinoembryonic antigen level of 2.4 ng/mL will be used as a marker to identify a malignant pleural effusion
  The cutoff level of 2.4 ng/mL was chosen based on a previous study, which showed a sensitivity of 93.5% and specificity of 73% at this threshold [16]

#### Study Population Recruitment

Patients satisfying the inclusion and exclusion criteria will be chosen using the nonprobability convenience sampling method.

#### Sample Size Calculation

The sample size was calculated based on the previously published study by ElSharawy et al [26], in which the mean CR in MPE was 52.43 (SD 19.36). Assuming 80% power and accepting a type-I error of 5%, the sample size was calculated to be 28 patients for this study.

The sample size for this study was calculated manually using the formula:



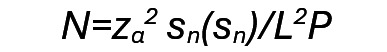



where *N* is the sample size, *z_α_*=1.96 at a 95% CI, *s_n_* is the sensitivity taken as 91% from a previous study [26], *L* is the precision of 15 %, and *P* is the expected prevalence of 50%. The precision is kept at 15% so that the calculated sample size (N=28) is feasible enough to be completed in the stipulated study duration of 2 years.

### Data Collection

The study investigator will take written informed consent from admitted patients with pleural effusion who satisfy the inclusion and exclusion criteria outlined above. The study investigator will ensure that the aspirated pleural fluid sample is sent for protein, glucose, LDH, ADA, CEA, and pleural fluid differential white cell count analysis and that simultaneously a serum sample is collected for estimation of protein and LDH levels. Microbiological analyses, which include Ziehl-Nelsen stain, Gram stain, ordinary culture, acid-fast bacillus culture of pleural fluid, and pleural fluid cytology, will be done in all the pleural fluid samples. Where available, reports of pleural biopsy, pleural fluid cell block, and pleural fluid GeneXpert Mycobacterium Tuberculosis/Rifampicin Ultra assay will also be used in the final data analysis.

Data extraction will be done manually by the study investigator, and details like clinicodemographic data of study participants, pleural fluid analysis reports, and other relevant investigations will be extracted from medical records using a study proforma developed by the authors as the data extraction tool, from which the results will be analyzed at the end of the study.

### Statistical Analysis

The analysis will be done by using the method of descriptive statistics. Statistical package IBM SPSS Statistics for Windows, version 23.0 (IBM Corp) will be used for the analysis. The actual *P* value will be recorded for all observations, and *P*<.05 will be considered statistically significant.

The diagnostic performance of CR and pleural fluid CEA as predictors for MPE will be compared using the receiver operating characteristics and the area under the receiver operating characteristics (area under the curve), taking cutoff values of >20 and 2.4 ng/mL, respectively.

A diagnostic 2×2 contingency table will be used to compare the performance of CR (at a cutoff of 20) and CEA (at a cutoff of 2.4 ng/mL) in arriving at the final diagnosis (as per methodology prespecified in the operational definitions).

### Ethical Considerations

The protocol is approved by the Institutional Ethics Committee, Kasturba Medical College, Mangalore, Manipal Academy of Higher Education, Manipal, Karnataka, India (IEC KMC MLE 02022/179) in May 2022. We will conduct this trial following the principles described in the Declaration of Helsinki. Written informed consent will be obtained from all participants before their inclusion in the trial.

## Results

Data collection began in June 2022. The expected date for data collection is between June 2022 and June 2024. As of March 24, 2024, we have recruited 22 patients. We anticipate completing the data analysis by August 2024. We expect the results to be published by January 2025.

## Discussion

### Principal Findings

This study primarily explores the use of CR in differentiating tuberculosis and malignancy as the cause of an exudative lymphocytic pleural effusion in hospitals without access to invasive investigations like thoracoscopy. This ratio is based on serum LDH and pleural fluid ADA measurements, which are simple, inexpensive, and point-of-care tests available at most primary health care centers.

### Strengths and Limitations

A significant strength of this study is that the prospective enrollment of patients will ensure that data collection will be complete in all aspects for most patients. This study design is better than a record-based retrospective study, which may not retrieve all parameters of research interest. Also, a single-center study within a stipulated time frame of 2 years ensures uniformity in detection methods and reagent kits used in the measurement of LDH, ADA, and CEA, which ensures the validity of the results. However, there are several limitations that the investigators envisage in this study. First, the study population is restricted to hospitalized patients who might have a higher LDH ratio due to underlying ailments, which could give a false positive CR even in patients without underlying malignancy. Second, the literature review reveals different cutoff values of the CR, with some studies showing an increase with age [28,29], whereas this study uses the most cited single cutoff value for the CR. If such variability is observed in this study, the authors plan to perform subgroup analysis using a logistic regression model to find the association between the confounding variable and the CR. Third, the relatively small sample size of this study may make it difficult to determine the association of histologic types of lung cancer to CR positivity in patients with MPE. Fourth, since lymphocytic exudates other than tuberculosis and malignancy are excluded in this study, we cannot be confident that the results may not also be seen in conditions such as rheumatoid pleuritis, benign asbestos effusion, and so on, which present with lymphocytic effusions.

### Future Research

Considering the wide range of cutoffs reported in the literature, further studies will be needed to validate the optimal standardized cutoff for CR in the Indian population. The sample size for a multicenter external validation of the optimum CR cutoff derived in this study is estimated to be 252 based on a precision of 5% and previous power analysis. Also, further research on CR in transudates and nonlymphocytic pleural effusions would be required before the findings of this study could be generalized to all MPEs.

### Conclusions

If found to be an accurate diagnostic test for MPE, CR has the potential to be used in community health settings and low-income countries without firsthand access to cytology or biopsy services. In such resource-limited settings, it could be used as a screening test for MPE, so that patients with high CR in their pleural fluid could be subsequently referred to centers where other invasive investigations to confirm an MPE, like a closed pleural biopsy, thoracoscopic biopsy, or image-guided (either computed tomography or ultrasound) biopsy, could be planned.

## Abbreviations

ADA: adenosine deaminase
CEA: carcinoembryonic antigen
CR: cancer ratio
LDH: lactate dehydrogenase
MPE: malignant pleural effusion
TPE: tuberculous pleural effusion

## Data Availability

The data that will support the findings of this study will be derived from patient medical record data. The datasets generated during and/or analyzed during this study are available from the corresponding author on reasonable request.

## References

[1] Ozcelik N, Ozcelik AE, Guner Zirih NM, Selimoglu I, Gumus A (2023). Deep learning for diagnosis of malign pleural effusion on computed tomography images. Clinics (Sao Paulo).

[2] Kumar Bar P, Mandal S, Banik T, Barman R, Mandal A (2019). A clinicopathological study of pleural effusion with special reference to malignant etiology in a tertiary care hospital in West Bengal. Int J Med Res Rev.

[3] Golwalkar JK, Shivpuje AV, Khandekar SV, Patil RS, Chahal M (2020). Study on etiology and clinical profile of pleural effusion. Int J Health Clin Res.

[4] Gupta R, Gupta A, Iliyas M (2018). Spectrum of pleural effusion etiology revisited in 18?70 years of age group: a tertiary care center-based study of 1000 patients. CHRISMED J Health Res.

[5] Kumar A, Kumar B, Verma Sk, Kumar A, Mathur Rk, Chaudhry S, Kant S (2020). A study to know the various causes of pleural effusion and role of pleural fluid adenosine deaminase enzyme in tuberculous pleural effusion. Int J Res Med Sci.

[6] Grippi MA, Antin-Ozerkis DE, Dela Cruz CS, Kotloff RM, Kotton CN, Pack AI (2023). Disorders of the pleural space. Fishman's Pulmonary Diseases and Disorders 6th ed.

[7] Gopi A, Madhavan SM, Sharma SK, Sahn SA (2007). Diagnosis and treatment of tuberculous pleural effusion in 2006. Chest.

[8] Light RW (2013). Pleural Diseases. 6th ed.

[9] Liang Q-L, Shi H-Z, Wang K, Qin S-M, Qin X-J (2008). Diagnostic accuracy of adenosine deaminase in tuberculous pleurisy: a meta-analysis. Respir Med.

[10] Sharma SK, Ryan H, Khaparde S, Sachdeva KS, Singh AD, Mohan A, Sarin R, Paramasivan CN, Kumar P, Nischal N, Khatiwada S, Garner P, Tharyan P (2017). Index-TB guidelines: guidelines on extrapulmonary tuberculosis for India. Indian J Med Res.

[11] Bibby AC, Dorn P, Psallidas I, Porcel JM, Janssen J, Froudarakis M, Subotic D, Astoul P, Licht P, Schmid R, Scherpereel A, Rahman NM, Cardillo G, Maskell NA (2018). ERS/EACTS statement on the management of malignant pleural effusions. Eur Respir J.

[12] Kassirian S, Hinton SN, Cuninghame S, Chaudhary R, Iansavitchene A, Amjadi K, Dhaliwal I, Zeman-Pocrnich C, Mitchell MA (2023). Diagnostic sensitivity of pleural fluid cytology in malignant pleural effusions: systematic review and meta-analysis. Thorax.

[13] Porcel JM, Esquerda A, Vives M, Bielsa S (2014). Etiology of pleural effusions: analysis of more than 3,000 consecutive thoracenteses. Arch Bronconeumol.

[14] Korczynski P, Krenke R, Safianowska A, Gorska K, Abou Chaz M B, Maskey-Warzechowska M, Kondracka A, Nasilowski J, Chazan R (2009). Diagnostic utility of pleural fluid and serum markers in differentiation between malignant and non-malignant pleural effusions. Eur J Med Res.

[15] Cavaco MJ, Mateus L, Nunes A, Cordeiro R, Silvestre C, Ferra J, Duarte D, Cardoso C, Raimundo P, Domingos A (2022). Diagnostic value of the cancer ratio in pleural effusion- a retrospective study. Eur Respir J.

[16] Krishnan VG, Kunoor A, Keechilath P, Mehta AA (2021). Diagnostic utility of pleural fluid carcinoembryonic antigen in patients with exudative pleural effusion. Lung India.

[17] Alataş F, Alataş O, Metintaş M, Colak O, Harmanci E, Demir S (2001). Diagnostic value of CEA, CA 15-3, CA 19-9, CYFRA 21-1, NSE and TSA assay in pleural effusions. Lung Cancer.

[18] Riantawan P, Sangsayan P, Bangpattanasiri K, Rojanaraweewong P (2000). Limited additive value of pleural fluid carcinoembryonic antigen level in malignant pleural effusion. Respiration.

[19] Villena V, López-Encuentra A, Echave-Sustaeta J, Martín-Escribano P, Ortuño-de-Solo B, Estenoz-Alfaro J (1996). Diagnostic value of CA 72-4, carcinoembryonic antigen, CA 15-3, and CA 19-9 assay in pleural fluid. A study of 207 patients. Cancer.

[20] Porcel JM, Vives M, Esquerda A, Salud A, Pérez B, Rodríguez-Panadero F (2004). Use of a panel of tumor markers (carcinoembryonic antigen, cancer antigen 125, carbohydrate antigen 15-3, and cytokeratin 19 fragments) in pleural fluid for the differential diagnosis of benign and malignant effusions. Chest.

[21] Ryu JS, Lee HJ, Cho JW, Han HS, Lee HL (2003). The implication of elevated carcinoembryonic antigen level in pleural fluid of patients with non-malignant pleural effusion. Respirology.

[22] Verma A, Abisheganaden J, Light RW (2016). Identifying malignant pleural effusion by a cancer ratio (serum LDH: pleural fluid ADA ratio). Lung.

[23] Forkasiewicz A, Dorociak M, Stach K, Szelachowski P, Tabola R, Augoff K (2020). The usefulness of lactate dehydrogenase measurements in current oncological practice. Cell Mol Biol Lett.

[24] Zhang Y, Li X, Liu J, Hu X, Wan C, Zhang R, Shen Y (2021). Diagnostic accuracy of the cancer ratio for the prediction of malignant pleural effusion: evidence from a validation study and meta-analysis. Ann Med.

[25] Verma A, Dagaonkar RS, Marshall D, Abisheganaden J, Light RW (2016). Differentiating malignant from tubercular pleural effusion by cancer ratio plus (cancer ratio: pleural lymphocyte count). Can Respir J.

[26] ElSharawy DE, Hagras MM, Khedr R (2020). The clinical utility of joined detection of cancer ratio, cancer ratio plus, Interferon gamma (IFN-ϒ) add carcinoembryonic antigen (CEA) in differentiating lymphocytic pleural effusions. Egypt J Bronchol.

[27] Alemán C, Sanchez L, Alegre J, Ruiz E, Vázquez A, Soriano T, Sarrapio J, Teixidor J, Andreu J, Felip E, Armadans L, Fernández de Sevilla T (2007). Differentiating between malignant and idiopathic pleural effusions: the value of diagnostic procedures. QJM.

[28] Ren Z, Xu L (2021). Role of cancer ratio and other new parameters in the differential diagnosis of malignant pleural effusion. Clinics (Sao Paulo).

[29] Huang JH, Chen H, Zhang ZC, Gu J, Yan L, Jiang MP, Zheng WQ, Hu ZD, Jiang TW (2023). Age affects the diagnostic accuracy of the cancer ratio for malignant pleural effusion. BMC Pulm Med.

